# Supplementary material for: Content-rich biological network constructed by mining PubMed abstracts
Source: BMC Bioinformatics. 2004 Oct 8;5:147. doi: 10.1186/1471-2105-5-147 (PMC528731; doi:10.1186/1471-2105-5-147)
Supplement: Additional File 2 — The original results of the above study (non-essential files are deleted to keep the file size under the limit set by BMC bioinformatics). [file 1471-2105-5-147-S2.bz2 › chilibotAdditionalFile2/dip40/159ID7685215E561/html/JUN_TBP.html]

 


 **JUN** and **TBP** 
  
Found 81 abstracts in PubMed, retrieved 40.  
 

 What does Google say? 
 PDF only 
| .edu only 

---

**Interactive relationship** (e.g. stimulation, inhibition, etc)

**Stimulatory relationship**- More importantly, expression of a constitutive active MAP kinase kinase 6 Glu resulted in the phosphorylation of a His  **TBP**  fusion protein and increased direct interaction of  **TBP**  with c  **Jun** .  Ref: 11454854 J Biol Chem, 2001
- BaP  [ **TBP** ]  increased the DNA binding of nuclear proteins to the AP 1  [ **JUN** ]  consensus recognition element in liver, but decreased DNA binding in kidney slices.  Ref: 9852999 Toxicol Appl Pharmacol, 1998
**Neutral relationship**- Transcriptional activation by c  **Jun**  depends on the TATA binding protein  [ **TBP** ]   **TBP**  associated factor TAF subunits of transcription factor IID TFIID  [ **TBP** ] .  Ref: 11316804 J Biol Chem, 2001
- These findings suggest that in macrophages, the p38 MAP kinase regulates AP 1  [ **JUN** ]  driven transcription by modulating the activation of  **TBP** .  Ref: 11454854 J Biol Chem, 2001
- Protein protein interaction assays revealed that c  **Jun**  binds with high specificity to the largest subunit of human TFIID  [ **TBP** ] , TAF II 250.  Ref: 11316804 J Biol Chem, 2001
- To examine protein protein interactions with cellular proteins at the visna virus promoter, we used an in vitro protein affinity chromatography assay and electrophoretic mobility shift assay, in addition to an in vivo two hybrid assay, to show that the visna virus Tat protein specifically interacts with the cellular transcription factors Fos and  **Jun**  and the basal transcription factor  **TBP**  TATA binding protein  [ **TBP** ] .  Ref: 9847304 J Virol, 1999
- Possible regulatory binding sites for general transcription factors, Sp1 and AP1  [ **JUN** ] , were mapped in the 5 promoter regions of CYP28B1 whereas TFIID  [ **TBP** ]  and Oct 1 were mapped in CYP4G13v2.  Ref: 12044492 Insect Biochem Mol Biol, 2002
- The results of EMSA showed that c  **Jun**  overexpression produced a binding of nuclear factor, which was HEK cell specific and distinct from TATA box binding protein  [ **TBP** ]  and AP 1  [ **JUN** ]  family transcription factor, to the renin core promoter region RC element from 36 to 20.  Ref: 9690204 Kidney Int, 1998

**Non-interactive relationship** (e.g. studied together, co-existance, homology, etc.)

- We hypothesized that c  **Jun**  is capable of derepressing the effect of the TAF II 250 N terminus on TFIID  [ **TBP** ]  driven transcription.  Ref: 11316804 J Biol Chem, 2001
- HBx upregulates the activity of a number of transcription factors including NF kappa B, AP 1  [ **JUN** ] , CREB, and  **TBP** .  Ref: 11325602 Cytokine Growth Factor Rev< MedlineTA>Cytokine Growth Factor Rev, 2001
- These results suggest that acute BaP  [ **TBP** ]  challenge is associated with altered expression of several growth related genes and AP 1  [ **JUN** ]  signaling and establish precision cut slices as a useful in vitro system to investigate the molecular basis of BaP  [ **TBP** ]  induced tumorigenesis, including organ specific differences.  Ref: 9852999 Toxicol Appl Pharmacol, 1998
- The E7 protein interacts with transcription regulatory proteins such as the TATA box binding protein  [ **TBP** ]  and with proteins of the AP1  [ **JUN** ]  transcription factor family.  Ref: 10725427 J Gen Virol, 2000
- Structural analysis of the PAHX AP1  [ **JUN** ]  with three reported hBAI associated proteins BAP  [ **TBP** ]  revealed no homology among them, and we designated PAHX AP1  [ **JUN** ]  as BAP4.  Ref: 11245925 Brain Res Mol Brain Res, 2001
- To further understand the molecular mechanism of glucocorticoid action on gene expression, DNA binding activities of the cis acting transcription factors activator protein 1  [ **JUN** ]  AP1  [ **JUN** ] , AP2, Egr1 zif268, NF kappaB, the signal transducers and activators of transcription proteins gamma interferon activation site GAS, Sis inducible element, and the TATA binding protein  [ **TBP** ]  transcription factor II D TFIID  [ **TBP** ]  were examined in human epidermal keratinocytes.  Ref: 9520467 Proc Natl Acad Sci U S A, 1998
- The human UBF promoter lacks a TATA and CAAT boxes but contains multiple binding sites for SP1, AP1  [ **JUN** ] , AP2, TFIID  [ **TBP** ] , NF 1 and a single site for NFAT 1.  Ref: 11916260 DNA Seq, 2001
- In support of this hypothesis, we found that c  **Jun**  increased levels of TFIID  [ **TBP** ]  driven transcription in vitro when added at high concentrations to a DNA template lacking activator protein 1  [ **JUN** ]  AP 1  [ **JUN** ]  sites.  Ref: 11316804 J Biol Chem, 2001
- In the present study we have analysed the mechanism of c  **Jun**  function as a trans activator with respect to members of the basal transcription machinery, TATA binding protein  [ **TBP** ]  associated factors TAFs.  Ref: 12676957 J Biol Chem< MedlineTA>J Biol Chem, 2001
- DNA sequence analysis of the promoter revealed a number of consensus binding sites for known transcription factors such as SP1, AP1  [ **JUN** ] , NFkappaB, C EBP and TFIID  [ **TBP** ] , and a repeat GC GT sequence motif seen for the formation of Z type DNA.  Ref: 9565665 Biochim Biophys Acta, 1998
- In contrast, binding activity of the transcription factors TFIID  [ **TBP** ] , AP 1  [ **JUN** ] , and SP 1 was highest in activated HSCs and only barely detectable in resting early activated HSCs.  Ref: 10595913 Am J Pathol, 1999
- It probably exerts its oncogenic functions by interacting and altering the normal activity of cell cycle control proteins such as p21WAF1, p27KIP1 and pRb, transcriptional activators such as  **TBP**  and AP 1  [ **JUN** ] , and metabolic regulators such as M2 pyruvate kinase M2 PK.  Ref: 11127826 Oncogene, 2000
- To elucidate further the mechanisms leading to the deletion, we have sequenced the novel 0.5 kb region residing immediately 3 to the breakpoint and shown that it contains putative binding sites for several transcription factors, such as HNF 1, AP 1  [ **JUN** ] , and TFIID  [ **TBP** ] .  Ref: 9272169 Hum Genet, 1997
- Scanning the BPV 1 genome for potential binding sequences, an intragenic AP 1  [ **JUN** ]  site BAP  [ **TBP** ]  1 within the E7 open reading frame was detected.  Ref: 12867421 J Biol ChemJ Biol Chem, 1997
- Electrophoretic mobility shift assays carried out to investigate its DNA binding properties established that trc S binds to HBV X promoter and oligonucleotides representing binding sites for the AP1  [ **JUN** ]  and TFIID  [ **TBP** ]  transcription factors.  Ref: 10745225 J Med Virol, 2000
- It is the regulatory subunit of the DNA dependent protein kinase that phosphorylates many proteins, including SV 40 large T antigen, p53, RNA polymerase II, RP A, topoisomerases, hsp90, and many transcription factors such as c  **Jun** , c Fos, oct 1, sp 1, c Myc, TFIID  [ **TBP** ] , and many more.  Ref: 10755664 Crit Rev Biochem Mol Biol, 2000
- Moreover, c  **Jun**  blocked the repression of  **TBP**  DNA binding caused by the N terminus of TAF II 250.  Ref: 11316804 J Biol Chem, 2001
- 5 FU treatment did not influence the activities of AP 1  [ **JUN** ] , AP 2, Oct 1, SP 1, CRE B and TFIID  [ **TBP** ] .  Ref: 12584750 Int J Cancer, 2003
- E7 is capable of recognizing a variety of proteins, including RB105, TATA box binding protein  [ **TBP** ]   **TBP** ,  **TBP**  associated factor TAF II 110, E2F, cyclins A and D, and c  **jun** .  Ref: 10881991 J Hum Virol< MedlineTA>J Hum Virol, 2003
- Finally, we show that SPI B interacts with the PU.1 cofactors Pip,  **TBP** , c  **Jun**  and with lower affinity to nuclear factor interleukin 6beta and retinoblastoma.  Ref: 10196196 J Biol Chem, 1999
- Strikingly, the first modification observed is methylation of histone H3 lysine 4, which correlates with the binding of the SET9 methyltransferase and the assembly of a complex consisting of c  **Jun** , c Fos, TATA binding protein  [ **TBP** ] , and RNA polymerase II.  Ref: 12588998 Mol Cell Biol, 2003
- EMSA assay resulted in the detection of five functional consensus sequences respectively for the transcription complex TFIID  [ **TBP** ]  and for the transcription factors AP 1  [ **JUN** ]  one site, AP 2 one and Sp1 two.  Ref: 11979512 Cell Biochem Funct, 2002
- Binding of activator protein 1  [ **JUN** ]  to DNA was enhanced in BaP  [ **TBP** ]  treated cells, but this change did not involve truncation or mutation of the c  **jun**  delta region.  Ref: 12106603 Biochem Pharmacol, 2002
- To investigate the molecular events leading to apoptosis induced by BaP  [ **TBP** ] , we studied the involvement of the interleukin 1beta converting enzyme ICE Ced 3 family of proteases caspases and c  **Jun**  NH2 terminal kinase 1 JNK1, which have been shown to mediate numerous extracellular stimuli induced apoptosis.  Ref: 9605752 Cancer Res, 1998
- We investigated the effects of black raspberry extracts on transactivation of activated protein 1 AP 1  [ **JUN** ]  and nuclear factor kappaB NFkappaB induced by BaP  [ **TBP** ]  diol epoxide BPDE, the ultimate carcinogen of BaP  [ **TBP** ] , in mouse epidermal JB6 Cl 41 Cl 41 cells.  Ref: 12460899 Cancer Res, 2002
- Testing of antisense treated LNCaP cells revealed that expression levels of other proteins that contain shorter polyglutamine sequence stretches such as HDAC2, TFIID  [ **TBP** ] , and c  **jun**  were not affected.  Ref: 11857028 Cancer Gene Ther, 2002
- The 5 flanking region contains potential binding sites for TATA binding protein  [ **TBP** ] , Sp1, nuclear factor 1 NF1, CAAT box binding protein C EBP, hepatocyte nuclear factors 1 and 5 HNF1, HNF5 and activator proteins 1 and 2 AP1  [ **JUN** ] , AP2.  Ref: 9305755 Gene, 1997
- c fos and c Ha ras gene expression was enhanced in liver, but not kidney, slices by BaP  [ **TBP** ] . c  **jun**  mRNA levels were decreased in liver and kidney slices, although the effect was earlier 0.5 h in liver slices compared to kidney slices.  Ref: 9852999 Toxicol Appl Pharmacol, 1998
- The association between Tat, Fos, and  **Jun**  would position Tat proximal to the viral TATA box, where the visna virus Tat activation domain could contact  **TBP**  to activate viral transcription.  Ref: 9847304 J Virol, 1999
- Chromatin immunoprecipitation analyses demonstrated that T cell activation triggers in vivo binding of endogenous c Rel, c  **Jun** , C EBP beta, and HMG IC to the bfl 1 regulatory region, coincident with selective recruitment of coactivators TAFII250 and p300, SWI SNF chromatin remodeling factor component BRG 1, and basal transcription factors TATA binding protein  [ **TBP** ]   **TBP**  and TFIIB, as well as hyperacetylation of histones H3 and H4.  Ref: 12665576 Mol Cell Biol, 2003
- We have isolated the 5 flanking region of the ok Npt2 gene including exons 1 3 and containing a TFIID  [ **TBP** ]  site, a GCCAAT site, an AP1  [ **JUN** ]  site, and two microsatellite GGAA repeats.  Ref: 9458840 Am J Physiol, 1998
- Enhanced dimerization of phosphorylated ATF 2 together with c  **Jun**  and binding to BAP  [ **TBP** ]  1 seems to be responsible for viral dysregulation, since both suppression of BPV 1 and c  **jun**  mRNA induction could be almost entirely abrogated by simultaneous treating with SB 203580, an inhibitor of p38 MAP kinase activity.  Ref: 12867421 J Biol ChemJ Biol Chem, 1998
